# Current Status of Gene Therapy in Hepatocellular Carcinoma

**DOI:** 10.3390/cancers11091265

**Published:** 2019-08-28

**Authors:** Saranya Chidambaranathan Reghupaty, Devanand Sarkar

**Affiliations:** Department of Human and Molecular Genetics, Massey Cancer Center, VCU Institute of Molecular Medicine (VIMM), Virginia Commonwealth University, Richmond, VA 23298, USA

**Keywords:** HCC, gene therapy, clinical trials, nanoparticles, viruses

## Abstract

Hepatocellular carcinoma (HCC) is the fifth most common cancer and the second leading cause of cancer related deaths world-wide. Liver transplantation, surgical resection, trans-arterial chemoembolization, and radio frequency ablation are effective strategies to treat early stage HCC. Unfortunately, HCC is usually diagnosed at an advanced stage and there are not many treatment options for late stage HCC. First-line therapy for late stage HCC includes sorafenib and lenvatinib. However, these treatments provide only an approximate three month increase in survival. Besides, they cannot specifically target cancer cells that lead to a wide array of side effects. Patients on these drugs develop resistance within a few months and have to rely on second-line therapy that includes regorafenib, pembrolizumab, nivolumab, and cabometyx. These disadvantages make gene therapy approach to treat HCC an attractive option. The two important questions that researchers have been trying to answer in the last 2–3 decades are what genes should be targeted and what delivery systems should be used. The objective of this review is to analyze the changing landscape of HCC gene therapy, with a focus on these two questions.

## 1. Introduction

### 1.1. Hepatocellular Carcinoma (HCC)

HCC arises from hepatocytes of the liver and it is the most common type of primary liver cancer. While improvements in health care have driven cancer related deaths drastically down in the United States (US,) HCC seems to be following a different trend. There was a 43% increase in liver cancer mortality in the US between 2000 and 2016 and, according to Globocan 2018, it is expected to increase another 41% by 2040. This prediction has been attributed to factors, like increasing obesity, diabetes, Non-alcoholic steatohepatitis, and an aging baby boomer population with Hepatitis C Virus infection [1]. Current chemotherapy treatments provide an increase in overall survival of only a few months, making it crucial to advance research and discover novel treatment options for HCC patients [2].

### 1.2. Gene Therapy

Gene therapy refers to the delivery of therapeutic nucleic acids into a person’s cells to cure a genetic condition. The nucleic acid can be delivered in vivo or ex vivo and by viral or non-viral means. In the context of cancer, some ways to correct abnormal gene expression are to introduce nucleic acids that code for a downregulated tumor suppressor gene, a small interfering RNA (siRNA) that prevents the translation of oncogenic mRNAs or suicide genes that produce molecules causing cancer cells to undergo apoptosis. Though there are in vivo and ex vivo FDA approved gene therapies for various conditions, only ex vivo cancer gene therapies have been approved so far. In August 2017, Tisagenlecleucel (trade name: Kymriah) became the first FDA approved gene therapy for acute lymphoblastic leukemia and diffuse large B-cell lymphoma (DLBCL) [3], and is closely followed by Axicabtagen ciloleucel (trade name: Yescarta) approved in October 2017 for DLBCL and Non-Hodgkins lymphoma [4].

## 2. Types of Gene Therapy

### 2.1. Ex Vivo Gene Therapy

Ex vivo gene therapy refers to the genetic manipulation of cells outside the body. In cancer, it involves removing cells from a patient, culturing and modifying them in a laboratory to give them the ability to destroy cancer cells, and delivering them back to the patient. Chimeric Antigen Receptor T (CAR-T) cells are the most commonly used cells. In this technique, T-cells from the patient are modified using a virus that codes for a gene that produces a receptor, called Chimeric Antigen Receptor (CAR), hence the name CAR-T cells. This receptor varies for each cancer type, is specific to cancer cells, and gives T cells the ability to specifically bind to cancer cells without harming healthy cells. Some of the targets used in CAR-T therapy are discussed below and Table 1 summarizes studies that use CAR-T cells for HCC treatment.

#### 2.1.1. Glypican-3 (GPC-3)

GPC-3 is the most commonly used CAR to target HCC cells. GPC-3 is a 70 kDa cell surface protein that belongs to the family of heparan sulfate proteoglycans. It attaches to the exocytoplasmic surface of the plasma membrane through a covalent glycosyl-phosphatidylinositol anchor. It binds to Wnt, Hedgehog proteins, and fibroblast growth factors and it signals for cell growth and differentiation [5]. It is required during embryonic development and it is not expressed in normal adult tissues. However, during pathological conditions, like cancer, its level increases. GPC3 is expressed in approximately 70% of HCCs, whereas it is not detectable in hepatocytes from normal liver and benign liver diseases. In many cases, there is no correlation between GPC3 and AFP values. Thus, at least one of the two markers is elevated in approximately 80% of HCC patients [6].

#### 2.1.2. Alpha Fetoprotein (AFP)

The molecular target for ET1402L1-CAR-T therapy is AFP. AFP is the most commonly used biomarker for HCC. It is a 70 KDa glycoprotein that is primarily found in the liver and the embryonic yolk sac of developing fetus and it acts as a transporter for molecules, like copper, nickel, bilirubin, and fatty acids. Serum AFP levels become almost undetectable after birth. However, it tends to rise during liver, ovarian, testicular cancers, and non-cancerous liver diseases, like cirrhosis and hepatitis. AFP is secreted in almost >80% of all HCC tumors and it is used as a serological marker for HCC diagnosis with other diagnostic tests [7].

#### 2.1.3. Cluster of Differentiation 147 (CD147)

CD147 is a 70 KDa transmembrane glycoprotein, which is highly expressed on the surface of various cancer cells. It contributes to HCC by affecting metabolic reprogramming, cellular proliferation, invasion and motility [8,9]. It also has a role in radio-resistance [10]. CD147 was shown to be expressed in HCC cell lines, but not in a human normal hepatic cell line. Immunohistochemistry on patient tissues revealed that CD147 was localized to the membrane of tumor cells in 74% HCC patients. A higher CD147 expression correlates with patient survival and HBV infection status [9,10].

#### 2.1.4. Mucin-1 (Muc1)

Muc1 is a transmembrane protein that has heavy O-linked glycosylation in its extracellular domain. It is known to promote radio-resistance, migration, and invasion in HCC [11]. Knockdown of Muc1 significantly inhibits cell proliferation and it promotes cell-cell aggregation and apoptosis. It blocks the expression of cyclin D1 and c-Myc and enhances the expression of E-cadherin. In vivo assays demonstrated that mice that were injected with Muc1-silenced cells did not develop tumors [12]. Wisteria Floribunda agglutinin (WFA) is a probe that detects sugar-related changes in cholangiocarcinoma. WFA-positive sialylated Muc1 is a diagnostic marker for cholangiocarcinoma. In addition, serum WFA-sialylated Muc1 level indicates hepatic progenitor cells or biliary features in HCC [13].

#### 2.1.5. Epithelial Cell Adhesion Molecule (EpCAM)

EpCAM is a glycoprotein that is only found in epithelial cells and in cancers but not in normal hepatocytes. It is responsible for cell-cell homotypic adhesion of epithelial cells. Patients with a high expression of this protein have high AFP levels and poorer differentiation of the tumor [14]. Upon sorting, EpCAM- and AFP-double positive HCC cells showed features of hepatic stem or progenitor cells [15].

#### 2.1.6. NY-ESO 1

NY-ESO-1 is a protein that belongs to the family of cancer testis antigens that are restricted to testicular germ cells in normal adult tissues and expressed in a variety of malignant tumors. NY-ESO-1 mRNA is detected in approximately 25% HCC tissue samples [16,17]. HepG2 cells with stable transfection of NY-ESO1 show an increase in migration as compared with control HepG2 cells. As a result of this cell migration, patients expressing high NY-ESO-1 might have worse HCC outcome following surgery [18].

### 2.2. In Vivo Gene Therapy 

In in vivo gene therapy, therapeutic nucleic acids are directly delivered inside a patient’s body using a viral or non-viral delivery system. The route of administration could be intravenous, intra-arterial, intra-tumoral, intra-portal, intra-splenic, or intra-biliary injection [19]. In vivo gene therapy has not been as successful as ex vivo gene therapy for cancer primarily because of the increased risk of unprecedented effects.

#### 2.2.1. Non-Viral Delivery Systems

##### Nanoparticles (NPs)

NPs are nanometer sized (<100 nm) colloidal particles that are used for the intracellular delivery of therapeutic agents. Nanoparticles are safer than other in vivo therapies, because there is no permeant genetic modification involved. Nanoparticles enter blood vessels, are taken up by cells through endocytosis, released from the endosome, release the siRNA, and the siRNA blocks the target RNA from translation by target mRNA degradation. ALN-VSP is a Lipid NP (LNP) that is a combination of Vascular Endothelial Growth Factor (VEGF) siRNA and kinesin spindle protein (KSP) siRNA in a ratio of 1:1. The intravenous administration of ALN-VSP to mice with orthotopic liver tumors caused a dose dependent decrease of both VEGF and KSP mRNA. It also caused a reduction in tumor hemorrhage and microvascular density and a 50% improvement in median survival as compared to control animals. Following these positive results, the safety, tolerability, pharmacokinetics, and pharmacodynamics of intravenous ALN-VSP was tested in a phase 1 clinical trial. It was found that the nanoparticle was generally well tolerated. The adverse event profile compared favorably with chemotherapy and other regularly used targeted therapies in cancer treatment. One patient, who had nodal and extensive liver metastasis, showed complete response. Patients who had nodal and extensive liver metastases showed prolonged disease stabilization for about 1 to 1.5 years. This is the only in-human RNA interference gene therapy trial [20].

(1) Nanoparticles Used as Monotherapy In Vitro

Some of the studies that have used nanoparticles to treat HCC are given below. However, it is to be noted they have all been done in HCC cell lines and/or animal models and have not yet moved into clinical trials. The characteristics, mechanism of action and target genes of nanoparticles are described in Table 2.

Chitosan is made from chitin, a naturally occurring substance that is found in shells of shrimp and other crustaceans by deacetylation. It is a polysaccharide that is cationic, biocompatible, biodegradable, and non-toxic. Hence, it is a suitable molecule for therapeutic RNA delivery [21]. Lipids are also advantageous for RNA delivery in vitro and in vivo because of their biocompatibility and biodegradability. Solid lipid nanoparticles (lipid monolayer enclosing a lipid core) and liposomes (lipid bilayer enclosing an aqueous core) are both used for RNA delivery. Chitosan-lipid nanoparticles combine the advantages of both these systems [22]. A lipid-based nanoparticle was used to deliver sphingosine kinase 2 (Sphk2) siRNA to the HCC cell lines and the xenograft mouse model. siRNA loaded nanoparticles were produced from (2E)-4-(dioleostearin)-amino-4-carbonyl-2-butenoic (DC) and chitosan [23]. MixNCH is a novel gene delivery vector that is synthesized by hybrid-type modification of chitosan with 2-chloro-ethylamine hydrochloride and N, N-dimethyl-2-chloroethylamine hydrochloride. Midkine siRNA was added to MixNCH to create MixNCH/MK-siRNA. This system was developed based on the hypothesis that the introduction of amino residues to chitosan could improve its solubility and stability to form a complex with negatively charged siRNA [24]. Asialoglycoproteins (ASGPRs) are carbohydrate binding proteins that are highly expressed on hepatocytes. Galactosylation of chitosan helps target nanoparticle to the liver [25]. Polyethylene glycol (PEG) has stealth properties that prevent its rapid elimination from circulation [26]. Therefore, Galactosylated chitosan-graft-poly (ethylene glycol) nanoparticles was used to deliver Polo-like Kinase 1 (PLK1) siRNA [27]. All of these siRNA-LNPs decreased cancer phenotypes in vitro and/or in vivo.

A lipid solution was made by mixing a positively charged lipid, a helper lipid, neutral lipid, and a PEG lipid in order to produce RNA-lipid nanoparticles (siRNA-LNPs) against Yes-Associated Protein (YAP), and the solution was mixed with YAP siRNA. The neutral lipid, Disteroylphosphatidyl Choline (DSPC), increases the encapsulation efficiency of the liposome and the helper lipid, cholesterol, improves intracellular delivery and LNP stability in vivo [28]. These nanoparticles caused a de-differentiation of HCC cells and tumor regression in an in vivo model. The YAP target levels are only increased in HCC patients that express a proliferative signature and absence of catenin beta 1 mutations. Thus, these siRNA-LNPs potentially work for this specific HCC subtype [29]. siRNA-LNPs have been used against Yes-Associated Protein (YAP) [29] and integrin [30]. LNPs enclosing miR-122 mimic have been used to restore levels of this tumor suppresser gene [31]. Both these studies reduced tumor characteristics in vitro and in vivo.

Superparamagnetic iron oxide nanoparticles (SPIOs) are cationic nanoparticles displaying good biocompatibility. SPIOs can be attached to nucleic acids and used for targeted gene transfer. SPIOs carrying VEGF siRNA with radiolabeled iodine 131 (131I) were used to treat nude mice with HCC. The tumors that were treated with nanoparticles grew nearly 50% slower than the controls [32]. Using the tripeptide arginine-glycine-aspartic acid (RGD) a magnetic resonance imaging-visible nanoparticle was generated. The surface of HCC cells contains certain integrin receptors that are not found in normal hepatocytes. RGD recognizes one such integrin receptor. Thus, RGD nanoparticles can specifically target tumor cells and deliver the therapeutic RNA. Polyethylenimine (PEI) has functionalized amine groups that facilitate the complexion and delivery of nucleic acids. An RGD-modified polyethylene glycol-grafted PEI, functionalized with SPION (RGD-PEG-g-PEI-SPION), was constructed for the delivery of Survivin siRNA. These nanocarriers were able to transfect HCC cells more efficiently than PEG-g-PEI-SPION, which is untargeted gene delivery. Tumor cell apoptosis was induced in vitro, and tumor growth was inhibited in vivo [33].

A NP that was made of an iron oxide core was used to target HCC cells by adding a monoclonal antibody against human GPC3 receptor. The nanoparticle core was coated with chitosan-PEG grafted PEI copolymer and functionalized with luciferase siRNA. The effective suppression of Luc expression was observed in vitro and in vivo [34]. A *Pyrococcus furiosus* ferritin nanocage containing the chemotherapeutic drug doxorubicin and the hepatocyte-targeting SP94 peptide decreased subcutaneous and metastatic HCC in vivo [35].

(2) Nanoparticles Used for Combination Therapy in Vitro

In vitro studies have tested nucleic acid encapsulated nanoparticles in combination with other HCC drugs. Some examples of these studies are as follows. A nanocarrier that specifically targets the liver (PAMAM-PEG-Gal) was synthesized by conjugating one end of PEG to poly(amidoamine) (PAMAM) dendrimer and the other end to galactose. AEG-1 siRNA molecules were complexed to this carrier to form PAMAM-AEG-1si. PAMAM dendrimers are molecules that have repetitive branches of amidoamine that radiate from a central core of ethylenediamine. Primary amines that are present on the surface impart a positive charge to these dendrimers enhancing binding to nucleic acids to be delivered and the cell surface. The treatment of orthotopic xenograft mice with PAMAM-AEG-1si resulted in a decrease in tumor growth. As compared to animals that were only treated with siRNA, animals treated with a combination of PAMAM-AEG-1si and all-trans retinoic acid showed an increase in necrosis and apoptosis and inhibition of proliferation [36].

A nanoparticle targeting ASGPR carrying sorafenib and siVEGF and synthesized from mesoporous silica nanocarrier (MSN) was tested for its effect in vitro. MSN has the advantage that its morphology, pore size, pore volume, and particle size can be controlled during synthesis [37]. This nanocarrier was produced by loading sorafenib onto MSN-NH_2_ nanoparticles, coating it with lactobionic acid (LA), and finally coating it with siRNA. HuH-7 cells that were transfected with this nanocarrier showed an increase in cell cycle arrest, enhanced toxicity, and improved tumor targeting by sorafenib and siVEGF. In another study, milk derived nanovesicles with small interfering RNA have been used to target β-catenin and enhance the therapeutic response to anti-PD1 therapy in transgenic HCC mouse model [38]. A hepatocyte specific LNP that uses SP94 to target liver cells was used to deliver both sorafenib and midkine to HCC cells. The LNP showed an increased cytotoxicity of HCC cells in vitro [39].

Cisplatin is a chemotherapy drug that is used to treat different cancers, including HCC, by inducing cell damage and apoptosis. However, advanced HCC patients can become resistant to HCC treatment. Combination therapy is used to solve this problem. Pt(IV))MNP/siNotch1 is a nanocarrier that co-delivers siRNA against Notch1 and the platinum(IV) prodrug to treat HCC. The prodrug, upon intercellular activation by reductive elimination, produces the active drug, Pt(II), which causes an inhibition in the proliferation and increase in apoptosis. Notch1 suppression increased the sensitivity of HCC cells to platinum drugs and decreased the percentage of HCC CSCs in HCC cells and a xenograft HCC model [40]. Another cationic cisplatin nanocapsule that was loaded with Bmi1 siRNA was produced by electrostatic complexation of siRNA and nanoparticles. The nanocapsule had cores that were composed of cisplatin and coated with cationic lipids. Treatment with these nanocapsules reduced the survival and proliferation of HCC cancer stem cells and eliminated stem cells in HCC mice models [41]. A PEI-modified MSN was used to deliver HNF4-alpha plasmid and cisplatin. Cisplatin acts on the entire tumor mass, whereas HNF-alpha plasmid decreases the stemness of HCC cells in vitro. The NPs were also able to suppress tumor growth in vivo [42].

##### Virus-Like Particles (VLPs)

VLPs are nanoparticles that resemble viruses in their structure, but they do not contain any viral genetic material. MS2 VLPs resemble the MS2 bactriophage. Its capsid can package and protect the target nucleotide from nuclease degradation. It has been used to specifically target HCC cells and deliver a variety of therapeutic agents. MSP VLPs with a modified peptide (SP94) have the ability to bind HCC cells with higher affinity than normal hepatocytes or other types of cells. SP94 modified MS2 VLP delivering a siRNA cocktail to inhibit cyclin expression was able to induce growth arrest and the apoptosis of Hep3B cells [43].

A novel delivery system was designed by cross-linking MS2 VLPs to GE11, an EGFR targeting peptide. These VLPs were able to bind to the EGFR receptor and deliver conjugated liposomes through the cell membrane without activating the receptor. This vector was tested for the delivery of a tumor suppressor, the long non-coding RNA (lncRNA) Maternally Expressed Gene 3 (MEG3) RNA, to EGFR+ HCC cell lines. These VLPs were fast, effective, safe, and attenuated tumor growth both in vitro and in vivo [44]. Cross-linking MS2 VLPs containing miR-122, to the cell penetrating protein, HIV Trans-Activator of Transcription (TAT), resulted in another novel delivery system. miR-122 is a tumor suppressor gene and human hepatmoa cells that were treated with MS2-TAT-miR122 VLPs were able to inhibit proliferation, migration, and invasion and induce apoptosis and suppress downstream target proteins. These results were also observed in in vivo studies [45].

#### 2.2.2. Viral Delivery

##### Adeno and Adeno Associated Virus

Adenoviruses are double stranded DNA viruses. Adenoviruses (AVs) and Adeno-Associated Viruses (AAVs) are both vectors that are used in gene delivery and can infect dividing and non-dividing cells without integrating with the genome of the host. AV has a complex 150 MDa capsid that surrounds its genome and core proteins. The capsid is icosahedral and made up of three major proteins, called hexon, fiber, and penton. Hexon is the most abundant protein on the capsid. There are 240 homotrimeric hexons on the capsid edges. Each vertex has one homopentameric penton base attached to a homotrimeric long fiber. During AV infection, the fibers bind to cell surface receptors, like CAR or CD46. Once the virus is bound to the cell surface, endocytosis occurs, and the virus enters the cell enclosed within vesicles. The virus is released from the endosome by endosome acidification. The viral DNA then enters the nucleus and is replicated to produce new viral proteins [46].

Human adeno virus 5 is the most commonly used virus in gene therapy. When an adenovirus infects a normal cell, it encodes a protein called E1B, which inactivates the tumor suppressor p53, which usually acts as a checkpoint and it prevents cells from going into the S phase if a viral genome is detected. This prevents the viral genome from replicating. A recombinant adenovirus lacking E1B and E3 (nonessential for either viral replication or infection) will not be able to replicate in a normal cell. p53 protein would recognize the viral DNA and halt S phase. In the case of a tumor cell, p53 is usually mutated. Hence, the viral genome replicates, which results in eventual cell death [47].

Most of the studies describing clinical trials are combination studies of recombinant AV with Transarterial Chemoembolization (TACE), Radiofrequency Ablation (RFA), or Hepatic Artery Infusion chemotherapy (HAIC). There are also studies that use rAd-p53. These are human adenoviruses with E1B and E3 removed and replaced with p53 coding sequence. When rAd-p53 is used in combination with TACE, viral particles of rAd-p53 are injected into the embolization artery. The most common adenoviral gene therapy strategy uses suicide genes, which will be discussed later. Table 3 summarizes clinical trials using adenovirus for HCC.

##### Vaccinia Virus

Vaccinia is a double-stranded DNA virus of the Poxviridae family. Pexastimogene devacirepvec; JX-594 (Pexa-Vec) is a targeted immunotherapeutic vaccinia virus with disruption of the viral thymidine kinase (TK) gene and a transgene for granulocyte macrophage colony-stimulating factor (GM-CSF). Viral replication is dependent on high cellular thymidine kinase activity and EGFR signaling that are both hallmarks of cancer cells. Thus, Pexa-Vec selectively infects and amplifies within the tumors. The infection of tumors by virus induces an adaptive immune response [48]. A phase I clinical trial (NCT00629759) in patients with primary or metastatic liver cancer was conducted to determine the maximum-tolerated dose and safety of JX-594 treatment. 14 of 22 patients met the inclusion criteria and they were included in the study. It was found that the maximum tolerated dose for intratumoral injection of JX-594 was 10^9^ pfu. JX-594 replication, GM-CSF expression and systemic dissemination were used as factors to assess the safety of JX-594 treatment and acceptable safety was observed in the patients [49]. This was followed by an open-label, randomized study of intratumoral injection of JX-594 in patients with unresectable primary hepatocellular carcinoma [50]. Two doses of the virus were tested for safety and efficacy (NCT00554372). A phase 3 trial with an estimated enrollment of 600 patients is currently in progress (NCT02562755). Table 4 summarizes clinical trials while using JX-594 (Pexa-vec) for HCC.

##### Lentivirus

**Figure 1:**
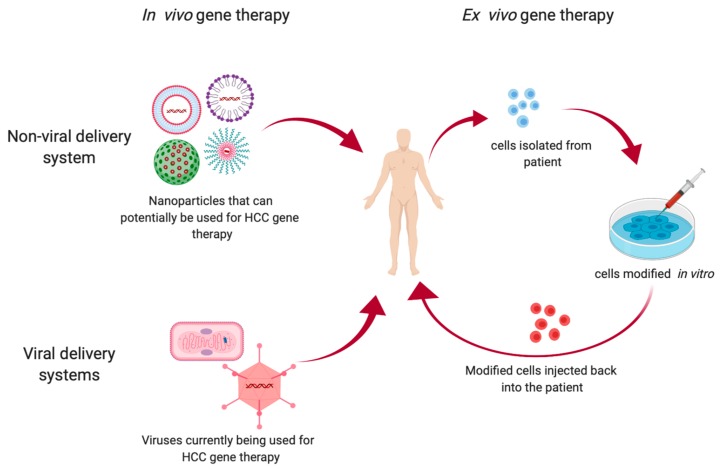
An overview of the types of gene therapy techniques used in HCC treatment.

Lentivirus belongs to the retroviridae family of viruses. Its genome consists of a single stranded RNA that is converted into double stranded DNA during replication. RNA is reverse-transcribed into DNA and is integrated into the host cell genome. After integration, the host cell transcribes the viral genes along with its own genes, which results in stable transgene expression. In addition, lentiviruses can infect both dividing and non-dividing cells, which makes them attractive for human gene therapy. Lentivirus is primarily used in gene therapy to engineer T cells in vitro in CAR-T therapy and to knock down cells in laboratory research [51,52,53,54]. Figure 1 provides a graphical representation of the types of gene therapy approaches.

## 3. Targets of Gene Therapy 

### 3.1. Suicide Genes

In suicide gene therapy, a transgene is delivered into the tumor and is expressed. Transgene delivery is followed by prodrug administration. The expression of the transgene converts the prodrug to a cytotoxic drug. However, there are practical difficulties to introducing a transgene to all cells in a tumor. This is resolved by the bystander effect, which is when the toxic product produced in one cell enters neighboring cells through cell-cell contact and helps in the complete elimination of cancer cells, even if all cells do not receive the transgene [55].

#### 3.1.1. Herpes Simplex Virus Thymidine Kinase/Ganciclovir System

HSVtk/GCV is the most commonly used suicide gene system. The conversion of GCV prodrug to its cytotoxic drug, GCV-triphosphate requires three phosphorylation reactions. The first phosphorylation by HSVtk produces GCV monophosphate. Two more phosphorylation reactions by different cellular kinases result in the production of GCV-triphosphate, a DNA chain terminator. Its incorporation during DNA replication results in cell death. This technique is usually used in combination with Liver Transplantation (LT). The first ADV-TK dose is injected into the peritoneum during surgery. Ganciclovir is slowly administered 36 hours after LT and once or twice after that. Another study has injected HSVtk intratumorally in patients with advanced hepatocellular carcinoma that were not amenable to curative therapy and administered ganciclovir later on.

#### 3.1.2. Cytosine Deaminase/5-Fluorocytosine System

Cytosine deaminase (CD) with the prodrug 5-fluorocytosine (5-FC) is another suicide gene therapy that has been studied in vitro. CD deaminates 5-FC into the cytotoxic drug 5-fluorouracil (5-FU), which is one of the standard drugs used in chemotherapy to treat HCC [56]. A combination suicide gene system using both CD and TK with a VEGF promoter was tested for HCC treatment in vitro and in vivo. VEGF is overexpressed in HCC cells, but not in normal hepatocytes. This only ensures prodrug activation in HCC cells. Individually, CD and 5-FC were cytotoxic, but their combination resulted in a significant decrease in the cell survival rate in vitro and angiogenesis and tumor growth in vivo as compared to their individual effect. [57].

#### 3.1.3. Purine Nucleoside Phosphorylase/ Fludarabine Phosphate System

Purine nucleoside phosphorylase (PNP) is an E. Coli enzyme that converts the prodrug fludarabine phosphate (FP) to the active drug, 2-fluoroadenine. Intracellular kinases then add phosphate groups to produce 2-fluoroadenine-triphosphate, which is an adenosine triphosphate analog. The incorporation of this analog during DNA and RNA synthesis halts the process and causes cell death. Only transfected cells produce the active drug, since human PNP gene cannot cleave fluarabine phosphate. HCC cells that were transfected with PNP gene while using ultrasonic nanobubbles showed a higher level of apoptosis when treated with fludarabine phosphate. The system also showed a notable bystander effect at low prodrug concentrations.

### 3.2. Oncogenes

Section Nanoparticles discusses studies that have used nanoparticles to treat HCC. siRNAs against Sphk2, Midkine, YAP, VEGF, survivin, Bmi, AEG-1, and Notch1 were chosen because extensive research has proved them to be potential targets.

#### 3.2.1. Midkine 

Midkine is a basic heparin-binding growth factor. It homodimerizes, is stabilized by heparin, and, as a result, constitutively activated. It is strongly induced by retinoic acid during mid-gestation but, found in very few adult cells. Midkine induces anoikis resistance, proliferation, and migration of HCC cells and tumor metastasis in mice [58,59]. It is one of the top five overexpressed genes in human HCC. Irrespective of AFP status, HCC patients display high serum midkine levels. Midkine can be used as a diagnostic and prognostic marker and a metastasis predictor [60].

#### 3.2.2. Yes-Associated Protein (YAP)

YAP expression is higher in HCC tissues as compared to adjacent normal tissue. Hypoxia dephosphorylates and causes nuclear translocation of YAP, hence activating it and promoting glycolysis. In the nucleus, active YAP interacts directly with and stabilizes HIF-1α to activate PKM2 transcription [61]. YAP promotes HCC metastasis and mobilization via governing cofilin/F-actin/lamellipodium axis by the regulation of JNK/Bnip3/SERCA/CaMKII pathways [62]. YAP is also a contributor to autophagy-related cell death and drug resistance in HCC [63].

#### 3.2.3. Survivin 

Survivin is an anti-apoptotic protein that belongs to the inhibitor of apoptosis protein family. It promotes cancer cell proliferation and tumor stromal angiogenesis, inhibits cancer cell apoptosis, and reduces radiation and the chemotherapy sensitivity of cancer cells [64]. It is also a prognostic marker [65]. In vitro studies have shown that treating HCC cell lines with a survivin suppressant, YM155, inhibits anchorage independent growth and induces cell cycle arrest and apoptosis. In an orthotopic liver tumor model that was treated with YM155 and sorafenib, YM155 treated animals showed lower tumor volume and an increase in apoptotic cells [66]. Tumor necrosis factor related apoptosis inducing ligand (TRAIL) is a cytokine that causes apoptotic cell death specifically in tumor cells. Survivin promotes the resistance of HCC cells to TRAIL treatment [67].

#### 3.2.4. B Cell-Specific Moloney Murine Leukemia Virus Integration Site 1 (Bmi-1)

Bmi-1 belongs to mammalian polycomb-group family and it is upregulated in early-stage HCC. The treatment of HCC cells with Bmi-1 shRNA decreases in vitro invasion, decreases the expression of MMP-2, MMP-9, VEGF, and p-Akt, and increases PTEN expression. Hence, it has been hypothesized that Bmi-1 could contribute to tumor metastasis by increasing MMP-2, MMP-9, and VEGF expression through the PTEN/PI3K/Akt pathway [68]. A similar experiment with shRNA mediated Bmi1 knockdown resulted in tumor growth inhibition in vitro and in vivo, proving that Bmi1 is a potential therapeutic target for HCC [69].

#### 3.2.5. Astrocyte Elevated Gene-1 (AEG-1)

AEG-1 gene is induced in primary human fetal astrocytes on HIV-1 infection or TNF-α treatment. In vitro and in vivo studies have shown that the overexpression of AEG-1 increases cancer phenotypes, like proliferation, invasion, migration, angiogenesis, metastasis, and chemo-resistance. Similarly, the knockdown of AEG-1 decreases all of these cancer properties [70]. It activates oncogenic signaling pathways, like PI3K/Akt, EGFR/MAPK, Wnt/β-catenin, and NF-κB. It has a role in macrophage activation during HCC. AEG-1 targeting in both HCC cells and HCC-associated macrophages might help to treat HCC [71]. Staphylococcal Nuclease Domain-Containing Protein 1 (SND-1), an AEG-1 interacting protein, is also an oncogene and a good target for gene therapy that is based on in vitro and in vivo studies [72].

#### 3.2.6. Notch-1

Notch1 is a protein that is essential for normal embryonic development. A meta-analysis of seven studies with 810 patients in total was conducted in order to address the controversial relationship between Notch-1 expression and prognosis in HCC patients. It was found that higher Notch-1 expression might predict poor prognosis and higher chance of tumor metastasis. Suppressing Notch signaling while using a gamma secretase inhibitor decreases in vitro invasion via ERK1/2 pathway and down-regulating MMP-2, MMP-9, and VEGF signaling [73].

Other than the above-mentioned oncogenes that have already been targeted using nanoparticles, there are a lot of potential therapeutic targets that have shown effects in vitro and in animal studies. This list includes c-Met, which is a receptor tyrosine kinase and a ligand for hepatocyte growth factor (HGF) [74], Gankyrin that has a role in p53 and RB interaction [75], Hypoxia Inducible Factor-1alpha, a transcription factor that is involved in the hypoxic response of cancer cells, Silent Information Regulator 1 (SIRT1) [76], a deacetylase that is involved in transcriptional silencing and cell survival, indoleamine 2,3-dioxygenase, an immunosuppressive enzyme that mediates tumor immune escape [77], and Plasmalemmal Vesicle-Associated Protein (PLVAP), an endothelial cell-specific protein [78].

### 3.3. Tumor Suppressor Genes

#### 3.3.1. Long Non Coding and Micro RNAs

Long non-coding RNAs (lncRNAs) are non-coding RNA sequences of >200 bases in length. LnRNAs have a role in cancer pathology and they can act as both oncogenes and tumor suppressors. Tumor suppressive lncRNA on chromosome 8p12 (TSLNC8) is an intergenic lncRNA LINC00589. RNA-sequencing data analysis and subsequent experiments proved that TNSLN8 is down-regulated in HCC. A Kaplan–Meier plot showed that lower TSLNC8 expression correlates with poor overall survival. Hence, it can be used as a prognostic marker for HCC. The overexpression of TSLNC8 inhibited cellular proliferation, invasion, and metastasis, whereas TSLNC8 silencing increased these characteristics both in vitro and in vivo. A pull-down assay revealed STAT3 as a TSLNC8 interacting protein. The tumor suppressive effect of TSLNC8 is due to inactivation of the interleukin-6 (IL-6)/STAT3 signaling pathway [79]. Some other lncRNAs that have a role in HCC are PDPK2P [80], GAS8-AS1 [81], Fer-1-like protein [82], MIR31HG [83], GAS5 [84], EPB41L4A-AS2 [85], FTX [86], lncWDR26 [87], Linc-USP16 [88], MIR22HG [89], and AOC4P [90]. In addition to lncRNAs, microRNAs (miRNAs) also have a role as tumor suppressor genes. miR-21-5p [91], miRNA-214 [92], miRNA-9 [93], miRNA- 370 [94], miRNA-622 [95], miR-1247-5p [96], miR-154 [97], miR-195 [98,99], miR-31 [100], miR-30e [101] miR-504 [102] regulate FASLG, PDK2 and PHF6, HMGA2, PIM1, MAP4K4, Wnt3, ZEB2, YAP and AEG-1, HDAC2 and CDK2, JAK1-STAT3-vimentin signaling, and FZD7, respectively.

#### 3.3.2. Potassium Voltage-gated Channel Subfamily Q Member 1 (KCNQ1)

KCNQ1 is a protein that has a role in the formation of potassium channels. It is expressed in HCC cell lines and clinical specimens at a lower level than normal hepatocytes and normal human tissue samples. Bioinformatic analysis showed that KCNQ1 has a role in the Epithelial-Mesenchymal Transition (EMT) process. KCNQ1 overexpression decreased the invasion of HCC cells in vitro and slowed tumor metastasis in vivo. It inhibits Wnt/β-catenin pathway by interacting with and sequestering β-catenin in the plasma membrane [103].

#### 3.3.3. Protocadherin9 (PCDH9)

PCDHs are a group of calcium-dependent adhesion proteins that make up a major subfamily of the cadherin superfamily. The methylation of PCDH9 promoter, and hence its down-regulation, is observed in 22% of primary HCC tissues. This methylation is associated with a larger tumor size and a more pronounced intrahepatic dissemination in HCC patients. Restoring PCDH9 expression inhibited the proliferation of HCC cell lines via inducing cell cycle arrest at G0/G1 phase in vitro and xenograft tumor formation in vivo [104].

#### 3.3.4. Vacuolar Protein Sorting-Associated Protein 4A (Vps4A)

Vps4A is a lysosomal or endosomal membrane trafficking protein that has a role in formation of exosomes. A comparison of Vps4A expression between tumor tissues and adjacent normal tissues revealed that Vps4A is down-regulated in tumor samples. Lower Vps4a level is also associated with poorer prognosis and tumor progression. The overexpression of Vps4A inhibits tumor proliferation, migration, and invasion in vitro and tumor formation in vivo. Overexpression of Vps4A inactivates phosphatidylinositol-3-kinase/Akt signaling pathway and it disturbs miRNA uptake and secretion by exosomes [105]. Vps4A also has a role in releasing β-catenin from the exosome of HCC cells, leading to EMT inhibition [106].

#### 3.3.5. Catenin Alpha 3 (CTNNA3)

CTNNA3 is a protein that has a role in cell-cell adhesion. Microarray data analysis identified CTNNA3 as a gene that is down regulated in HCC. Both overexpression and knockdown studies showed that CTNNA3 has a role in inhibiting proliferation, cell cycle progression in G1-S, migration and invasion of HCC cells in vitro. It also decreases tumor growth in vivo. CTNNA3 decreases cellular proliferation by inhibiting Akt signaling and increasing the cell cycle inhibitor p21^Cip1/Waf1^. It decreases cell invasion by inhibiting Akt signaling and inhibiting MMP-9 [107].

## 4. Conclusions

The liver has two sources of blood—the hepatic portal vein and the hepatic artery. Portal vein carries venous blood that is rich in nutrients, from the intestines, spleen, stomach, and gall bladder to the liver. It accounts for about 75% blood flow of the liver. The other 25% of the liver’s blood supply comes from the hepatic artery, which brings oxygenated blood from the heart to the liver. The hepatic portal artery transports any intravenously delivered agent directly to the liver before it enters systemic circulation, providing a huge advantage for hepatocyte targeted treatments. The liver has less than 1% of dividing hepatocytes normally. Hence, adenoviral vectors and lentiviruses that can infect non-dividing hepatocytes are efficient systems for gene therapy.

Despite all of these advantages, gene therapy techniques that have entered clinical trials are limited namely, CAR-T, thymidine kinase, p53, rhAdV, and RNAi (two trials), and JX-594 (Pexa-Vec) gene therapy. Developments in small molecule inhibitors have been rapid as compared to gene therapy, probably because of the ethical questions surrounding gene therapy. Another disadvantage is that cancer has no one size fits all solution. For example, during the CAR-T technique, patients are chosen based on cell surface expression of the target ligand, like glypican, mucin 1 and AFP on the tumors. Numerous clinical trials are being conducted in the US for monogenic diseases, like hemophilia A. However, the fact that cancer is a polygenic and multifactorial disease makes it difficult to determine the one gene to be targeted. This might make combination treatments more efficient than standalone gene therapy. Table 1 and Table 3 reveal that China is leading in HCC clinical trials. Not just clinical trials, China is also a major contributor to basic HCC research. The most important reason is that China has more than 53% of the world’s HCC population and this disease burden is predicted to keep increasing [108].

Huge developments have been made in the field of gene therapy in the last few decades, the most notable of which is CAR-T therapy. However, in the field of HCC the pace has not been as much as with other conditions. However, numerous potential therapeutic targets are being identified through in vitro and in vivo studies. This review highlights the progress made so far and the potential to find a treatment for HCC using gene therapy strategy.

## Figures and Tables

**Table 1:** Data from clinicaltrials.gov as of 07.13.2019 showing clinical trials that use Chimeric Antigen Receptor T (CAR-T) cells to treat Hepatocellular Carcinoma (HCC).

Study Identifier	Biological Used	Study Status	Delivery Route	Clinical Trial Phase	Adjuvant Therapy Used	Country	Patients Enrolled
NCT03146234	GPC-3 CAR-T	Recruiting	Intravenous	N/A	-	China	HCC patients with relapsed or refractory cancer
NCT02905188	GPC-3 CAR-T	Recruiting	Intravenous	Phase 1	-	USA	HCC patients with unresectable, recurrent and/or metastatic cancer
NCT03084380	GPC-3 CAR-T	Not yet recruiting	Intravenous	Phase 1/2	TACE	China	HCC patients who cannot receive TACE with sorafenib treatment
NCT03884751	GPC-3 CAR-T	Not yet recruiting	Intravenous	Phase 1	-	China	Advanced HCC patients with no effective treatment
NCT03980288	GPC-3 CAR-T	Not yet recruiting	Intravenous	Phase 1	-	China	Advanced HCC patients intolerant to standard treatment
NCT02723942	GPC-3 CAR-T	Completed	N/A	Phase 1/2	-	China	HCC patients with non-diffuse HCC without extrahepatic metastasis or portal vein vascular invasion.
NCT02395250	GPC-3 CAR-T	Completed	N/A	Phase 1	-	China	HCC patients with relapsed or refractory cancer
NCT03198546	GPC-3 CAR-T	Recruiting	N/A	Phase 1	-	China	Advanced HCC patients
NCT02715362	GPC-3 CAR-T	Unknown	Transcatheter Arterial Infusion	Phase 1/2	-	China	HCC patients with unresectable advanced cancer
NCT03130712	GPC-3 CAR-T	Unknown	Intratumoral	Phase 1/2	-	China	Advanced HCC patients with persistent cancer after standard chemotherapy or surgery
NCT03993743	CD147-CAR-T	Active, not Recruiting	Hepatic Artery Infusion	Phase 1	-	China	Advanced HCC patients who have failed first and second lines of HCC treatment
NCT03349255	ET1402L1-CAR-T	Terminated	Intravenous	Phase 1	-	China	HCC patients with at least one measurable tumor
NCT02587689	Anti-MUC1 CAR-T	Unknown	N/A	Phase 1	-	China	HCC patients with relapsed or refractory cancer
NCT03013712	EpCAM specific CAR-T	Recruiting	Vascular intervention	Phase 1	-	China	HCC patients with relapsed or refractory cancer

**Table 2:** The characteristics, mechanism of action and target genes of nanoparticles.

Nanodrug Type	Classification/Examples	Characteristics	Mechanism of Action	Gene(s) Targeted
Lipid nanoparticles	Micelles	Lipid monolayer enclosing a hydrophobic core.	The therapeutic agent is entrapped within the hydrophobic or aqueous core. Once drugs reach the cell membrane, they are taken up by the cell through endocytosis. As pH in the endosome decreases, the therapeutic agent is released. In case of siRNA, it incorporates into the RISC complex and cleaves target mRNA.	YAP, Integrin, miR-122, Bmi1
	Liposomes	Lipid bilayer membrane surrounding an aqueous core.		
Dendrimers	PAMAM	Dendrimers are nanoparticles with repetitive branching. PAMAM dendrimers have repetitive branches of amidoamine radiating from a central core of ethylenediamine.	The structure of PAMAM contains cavities within the assembled molecule that can be exploited to carry therapeutic agents. They also have positively charged primary amines on their surface that allow binding of nucleic acids. Dendrimers that are chemically modified to be recognized by membrane proteins are internalized by endocytosis after interaction with surface protein. Otherwise they are internalized by non-specific endocytosis	siAEG-1
Polysaccharides	Chitosan	Polysaccharide made up of units of beta (1→4) linked glucosamine and N-acetyl glucosamine	Therapeutic nucleic acids can be adsorbed onto chitosan nanoparticles, complexed with chitosan or enclosed within the chitosan nanoparticles. Cellular uptake is through non-specific endocytosis or receptor-mediated endocytosis.	Sphk2, Midkine, PLK1
Iron oxide	_	Made up of γ-Fe_2_O_3_ and/or Fe_3_O_4_ particles	Iron oxide nanoparticles can be produced using many methods including coprecipitation of ferric and ferrous ions. They are usually coated with polymers and negatively charged nucleic acids are bound to their surface.	VEGF
Mesoporous silica nanoparticles	_	Silica nanoparticles with pores of 2–50 nm	Surfactants are mixed with silica precursors to form silica structures around the surfactant. The surfactant is then removed to produce MSN. Therapeutic agents can be loaded into these pores and delivered into cells. MSNs can be modified to target certain cells. They are internalized through receptor-mediated or specific endocytosis and released into the cytoplasm.	VEGF, siNotch1

**Table 3:** Data from clinicaltrials.gov as of 07.13.2019 showing clinical trials that use adenoviruses to treat HCC.

Study Identifier	Biological Used	Study Status	Delivery Route	Clinical Trial Phase	Adjuvant Therapy Used	Country	Patients Enrolled
NCT01869088	rhAdV type-5	Unknown	Arterial injection	Phase 3	TACE	China	Advanced HCC patients who cannot undergo surgery or local ablative therapy
NCT03790059	rhAdV type-5	Recruiting	Intraoperative injection	N/A	RFA	China	Patients with single HCC of diameter less than 3 cm
NCT03780049	rhAdV type-5	Recruiting	Hepatic artery infusion	Phase 3	HAIC	China	HCC patients with unresectable tumors
NCT00669136	AFP AdV	Terminated (Poor accrual)	Intramuscular	Phase 1	-	USA	HCC patients with locoregionally pre-treated cancer
NCT02202564	ADV-TK	Completed	Intraperitoneal	Phase 2	Liver Transplant	China	HCC patients who can undergo liver transplantation
NCT00300521	ADV-TK	Completed	N/A	Phase 2	Liver Transplant	China	Intermediate or advanced HCC patients who can undergo liver transplantation
NCT00844623	TK99UN (AdV with HsV-TK)	Completed	Intratumoral	Phase 1	N/A	Spain	HCC patients who cannot undergo curative therapy
NCT03313596	ADV-Tk	Recruiting	Intraperitoneal	Phase 3	Liver Transplant	China	Advanced HCC patients who can undergo liver transplantation
NCT02561546	p53	Unknown	Arterial injection	Phase 2	TACE	China	HCC patients with unresectable cancer
NCT02509169	P53	Unknown	N/A	Phase 2	TAE	China	HCC patients with unresectable cancer
NCT00003147	Ad5CMV-p53 gene	Terminated (Administratively complete)	Percutaneous	Phase 1	-	USA	HCC patients with unresectable cancer
NCT02418988	rAd-p53	Unknown	Arterial injection	Phase 2	TACE	china	HCC patients with unresectable cancer

**Table 4:** Data from clinicaltrials.gov as of 07.13.2019 showing clinical trials that use JX-594. (Pexa-Vec) to treat HCC.

Study Identifier	Biological Used	Study Status	Delivery Route	Clinical Trial Phase	Adjuvant	Country	Patients Enrolled
NCT00629759	JX-594 (Pexa-Vec)	completed	Transdermal injection	Phase 1	-	Korea	HCC patients resistant to standard treatment
NCT01636284	JX-594 (Pexa-Vec)	completed	Intravenous	Phase 2a	-	US and Korea	Advanced HCC patients who have not been treated with. sorafenib
NCT01171651	JX-594 (Pexa-Vec)	completed	Intravenous and intratumoral	Phase 2	sorafenib	Korea	HCC patients with unresectable cancer
NCT01387555	JX-594 (Pexa-Vec)	completed	N/A	Phase 2b	-	US	Advanced HCC patients intolerant to sorafenib
NCT00554372	JX-594 (Pexa-Vec)	completed	Intratumoral	Phase 2	-	US	HCC patients with unresectable cancer
NCT02562755	JX-594 (Pexa-Vec)	Recruiting	Intratumoral	Phase 3	sorafenib	US	Advanced HCC without prior systemic therapy
